# Morphology, anatomy and sleep movements of *Ludwigia sedoides*

**DOI:** 10.1007/s00114-023-01848-7

**Published:** 2023-05-15

**Authors:** Katharina Heyder, Christoph Neinhuis, Thea Lautenschläger

**Affiliations:** Institute of Botany, Technische Universität Dresden, Dresden, Germany

**Keywords:** Nyctinasty, Circadian rhythmic, Submersing, Endogenous oscillator, Onagraceae, Water plant

## Abstract

The diurnal motion of higher plants, responding to the alternation of day and night, known as nyctinastic movements or “sleep movements”, has been discussed frequently. We present the first description of the circadian rhythm of the water plant *Ludwigia sedoides* (Humb. & Bonpl.) H.Hara of the family Onagraceae, furthermore its morphology and anatomy. Our results indicate that the plant’s movements are endogenous, although environmental factors certainly have an influence. The majority of plants with nyctinastic leaf movements have a pulvinus, as the crucial part of the plant enabling this movement. Although the basal section of the *L. sedoides* petiole is not swollen, the tissue functions similarly to a pulvinus. It consists of a central conducting tissue with thick-walled cells, which is surrounded by thin-walled motor cells that can undergo visible shrinking and swelling. Thus, the tissue functionally corresponds to a pulvinus. Examinations of cellular processes, like measurements of the turgor pressure in the petiole, need to be evaluated in future studies.

**Supplementary Information:**

The online version contains supplementary material available at 10.1007/s00114-023-01848-7.

## Introduction

The genus *Ludwigia* L., belonging to the family of Onagraceae, contains numerous species growing on the banks of lakes and rivers, because of their preference for moist or flooded sites. Some species of the genus are predominantly aquatic (Rocha and de Melo 2020). This also includes *Ludwigia sedoides* (Humb. & Bonpl.) H.Hara as the only species of the section Humboldtia in the genus (Ramamoorthy 1979), sometimes wrongly referred to as *L. sedioides* (Missouri Botanical Garden 2023)*. L. sedoides* is a rooted macrophyte with submersed stems and floating leaves, forming rosettes on the water surface. It is evergreen and produces yellow flowers throughout the year. A study by Barbosa et al. from 2014 identified some environmental factors that correlate with the coverage of *L. sedoides*: the preference for shallow lagoons and floodplains, protected from wind. The natural distribution of *L. sedoides* extends from Mexico and Central America to Brazil, often in Neotropical reservoirs (Barbosa et al. 2014). While cultivating this species in the Botanical Garden of the Technische Universität Dresden, it has been observed that the leaf rosettes contract and (partially) sink below the water level at night. This raised the question of how and why *Ludwigia sedoides* is able to perform these movements.

The first written mention of an observation of diurnal movements in plants is from the fourth century BC: the daily leaf movements of the tamarind tree, *Tamarindus indicus*, were described (Satter and Galston 1973). At that time, plant movements were regarded as the result of specific external events, such as the position of the sun or external mechanical influences such as wind (Rivière et al. 2017). The biophysical mechanism that underlies the rhythms or the ecological significance of diurnal movements in plants was not yet scientifically investigated. Even though some questions remained open, there is much more knowledge about circadian rhythms of plants today.

Circadian movements include diurnal changes in metabolic activities, organ positions, growth and differentiation processes (Kadereit et al. 2014). One of these rhythms are the nyctinastic movements, also referred to as “sleep movements”, which are of interest in this context.

Nyctinastic movements include the folding of flowers and leaves at dusk (Sisodia and Bhatla 2018), which also applies to the movement of *L. sedoides*. The majority of plants with nyctinastic leaf movements have a pulvinus, the leaf hinge. In these cases, it is the crucial part of the plant which enables the movement. The principle of this motor organ is based on turgor changes. The turgor of cells on one site of the pulvinus increases during the opening of the leaf and decreases during closure. This region is called the extensor. The opposite region shows reverse changes in the turgor and is called the flexor. The positions of these two regions are not fixed with the function (Satter and Galston 1981). Recently, the chemical aspects of the mechanism of pulvini are often discussed (Ueda et al. 2001, 2019; Ueda and Nakamura 2007; Raeini-Sarjaz 2011).

The ecological importance of circadian rhythms in general is relatively apparent. Through the sessile way of life of plants, it is a necessary mechanism to adapt their physiological, developmental and reproductive processes to the time of the day or the season. The function of sleep movements is not yet fully understood and it is often discussed what selective advantages foliar nyctinasty has. Various hypotheses were put forth to explain these movements. Darwin suggested that nyctinasty improves the temperature relations of plants (Darwin and Darwin 1880). Others proposed that the movement helps to remove surface water from the leaves (Dean and Smith 1978) or prevents the disruption of photoperiodism by moonlight (Bünning and Moser 1969). Four of the common hypotheses were discussed by Minorsky (2019). Some of the contents were disproven. Others do not explain the movements of all plants, such as aquatic plants.

Therefore, Minorsky postulates a tritrophic hypothesis. He suggests that the foliar nyctinasty is an indirect defence against nocturnal herbivores, by facilitating the hunting of nocturnal carnivores and by restricting the foraging of herbivores.

Many scientists have studied circadian rhythms and also nyctinastic movements have already been discussed frequently. Nevertheless, there are some unresolved issues, especially when looking at a nyctinastic species in detail. As mentioned, little scientific information is available, and the diurnal movements of *Ludwigia sedoides* are not mentioned at all.

The present study deals with the movement of *Ludwigia sedoides* petioles in detail. First, the movement of the plant will be described, starting with the observed process of the movement. In this context, it is also examined whether the movement could be generated in the leaf joint. Therefore, the movement of a single leaf, the movement of the rosette without the lamina and the complete leaf is going to be observed. Then the movement over time is examined. With the help of time-lapse recordings, the duration of the motion is determined. To assess the external environmental conditions, which might trigger the movement, the durations were compared with light intensity. For some more detailed information, a darkening experiment was conducted.

Furthermore, the anatomy and morphology of *L. sedoides* will be described, mainly by microscopic investigations with particular emphasis on the petiole base, in order to possibly detect pulvini. Microscopic measurements in this part of the plant were carried out to detect differences between day and night.

## Material and methods

### Plant material

*Ludwigia sedoides* is cultivated in the Botanical Garden of the Technische Universität Dresden (51°02′36″N, 13°45′32″E; 117 m a.s.l.), Germany, in a small aquarium and during summer additionally in a big water basin of the so-called Victoria House. The plant with the IPEN-Number BR-0-BONN-1014 was originally collected near Manaus/Brazil by Josef Bogner from the Botanical Garden in Munich. The Botanical Garden in Dresden received the plant from the Botanical Garden in Bonn.

### Plant movement observations

The study of its movement took place in the observation period from 18 May 2020 to 31 December 2020. A digital camera (Nikon Coolpix P7100) was used for all photos documenting the plant positions. In order to observe the movement of the plant, a stop-motion camera (brinno TimeLapse Camera TLC200 Pro; one image per 20 s) was used. While filming the plant in the large water basin, the camera was put in a small glass container. This allowed filming the plant from the side underwater. To document the duration of the movement seen on the videos, the time at the start and end of the movement in the morning and in the evening was noted. In those video recordings, e.g., from September, when the plant was already moving when the first daylight allowed filming in the morning and if it was still moving while it was already dark in the evening, the time of the first visible light on the video in the morning or the complete darkness in the evening was noted. To compare the duration of the movement with the exposure of light, the collected data on the external brightness during the observation period was provided by the Botanical Garden. These data from the meteorological station (pyranometer CMP 3, Intelligente Meßtechnik und Automatisierung GmbH Potsdam, Germany) were measured in lux every 12 min (Appendix 4). For comparison, the average values of the individual days were used.

To examine which part of the plant is responsible for the movement, the laminae of the rosette were removed with a razor blade. The movement of the plant without the laminae was filmed as described.

In order to study whether the movement could be endogenous, additional illumination and permanent darkness experiments were performed. For the darkening experiment, a light-tight cardboard box was placed over the small aquarium in the greenhouse at daytime. The experiment was performed three times: with the box in place for 3, 6 and 24 h. For the lighting experiment, a lamp placed above the plant was turned on at night for 5 h. This experiment was conducted twice. The first time, a daylight lamp (Valoya B100 LE17003847) was used. The second time, an assimilation lamp (Valoya B200 LE17005203) was used, under which 25,000 lx could be measured at night. The reaction of the plant was filmed again with the stop-motion camera.

### Anatomy

To examine the anatomy of the plant, microscopic sections were made. For these microscopic images, a digital microscope (KEYENCE VHX-970F) and a Stereo microscope system (Olympus SZX16) were used. Most of the sections were made with fresh plant material. To detect differences in petiole anatomy, several complete rosettes were collected during day and night and subsequently placed in 100% methanol for fixation of the tissues (Neinhuis & Edelmann 1996).

### Measurements and statistical analyses

All measurements were made with microscopic images of the cross sections using the software of the above mentioned microscope (KEYENCE VHX-970F).

First, the areas of single cells in the petiole base (night and day preparations, abaxial and adaxial side) were measured. The collected data about the cell sizes were statistically analysed with the independent two-sample *t*-test. The test was conducted to detect changes in cell size at the adaxial and abaxial sides of the petiole base between night and day. Therefore, the mean values of each side per cut were used. A total of 26 cross-sections were measured, 13 sections each from day and night conditions. The *t*-test was chosen because the variables are independent of each other and normally distributed (Rudolf and Kuhlisch 2008). The test was conducted with the one-sided hypothesis that the cells at night are larger than the cells at day: ${H}_{1}: {F}_{1}\left(x\right)= {F}_{2} \left(x-\theta \right), \theta >0$. This results in the alternative hypothesis ${H}_{0}: {F}_{1}\left(x\right)= {F}_{2} \left(x-\theta \right), \theta \le 0$. A significance level of 5% was used for this.

For an additional comparison, the whole cross-sectional area of the adaxial and abaxial sides of the petiole base and the areas of the intercellular spaces of the 26 cross-sections were measured. Both measurements are discussed as proportional numbers: the areas of both sides in relation to the size of the whole cross section, and the intercellular spaces in relation to the corresponding side. The variables are independent of each other and not normally distributed. Therefore, a Mann–Whitney *U* test was chosen (Rudolf and Kuhlisch 2008). The test was conducted with the one-sided hypothesis that the abaxial side is larger at night and the adaxial side is larger at day: ${H}_{1}: {F}_{1}\left(x\right)= {F}_{2} \left(x-\theta \right), \theta >0$. This results in the alternative hypothesis ${H}_{0}: {F}_{1}\left(x\right)= {F}_{2} \left(x-\theta \right), \theta \le 0$. A significance level of 5% was used for this. For the data on the intercellular spaces, again a *t*-test was chosen because the variables are normally distributed and independent of each other. The test was conducted with the one-sided hypothesis that the intercellular spaces at the abaxial side are larger at night and the intercellular spaces at the adaxial side are larger at day: ${H}_{1}: {F}_{1}\left(x\right)= {F}_{2} \left(x-\theta \right), \theta >0$. This results in the alternative hypothesis ${H}_{0}: {F}_{1}\left(x\right)= {F}_{2} \left(x-\theta \right), \theta \le 0$.

## Results

### Description of the movement

#### General rhythm

**Fig. 1:**
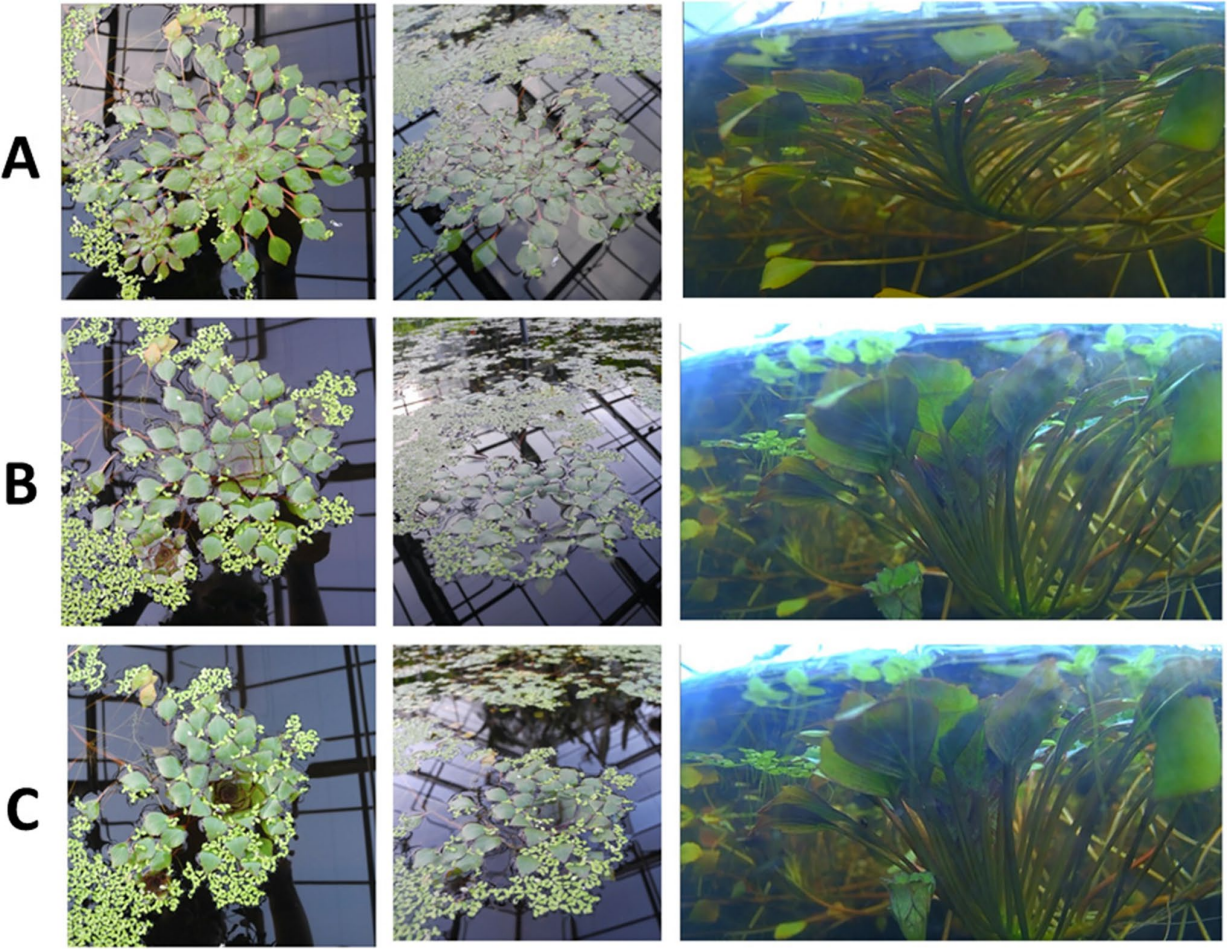
Movement of *Ludwigia sedoides* from above and sideways. **A** Spread rosette during day. **B** Submerged rosette in the middle of the movement. **C** Fully submerged rosette during night

At dusk, *Ludwigia sedoides* shows a distinct nyctinastic movement (Fig. 1). Throughout the day, the leaves of the rosettes are floating at the water surface (Fig. 1A). With the beginning twilight, the leaves move steadily towards the centre of the rosette, decreasing the diameter of the rosette, while the central leaves of the rosette become submerged (Fig. 1C). The plant shows this behaviour every evening while the rosettes spread again with the same extend of motion in the morning, starting with the first light.

**Fig. 2:**
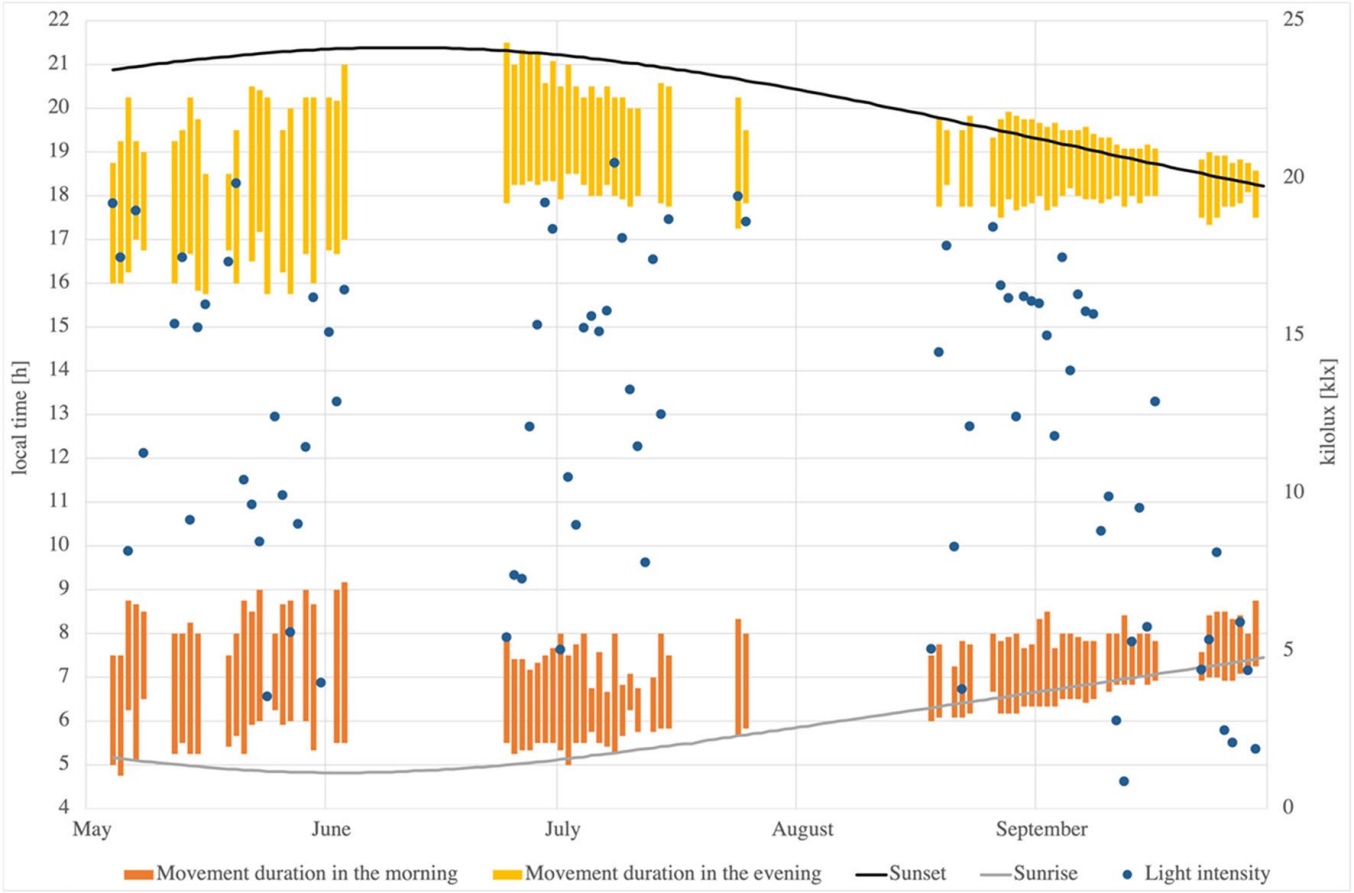
Duration of the movement of *Ludwigia sedoides* during the study period from 18 May 2020 to 13 October 2020. Yellow bars: duration of the movement in the evening. Orange bars: duration of the movement in the morning. Black line: time of sunset in Dresden (time and date AS n.d.). Grey line: time of sunrise in Dresden (time and date AS n.d.). Dots: daily average of external brightness in kilolux based on the data measured by the weather station at the Botanical Garden in Dresden, Germany

The data concerning the duration of the movement (Fig. 2) show that the evening movement, i.e., when the leaf rosette is closing, takes longer on average than the reverse movement in the morning. The difference of 27 min results from an average duration of 2.46 h in the evening and 2.01 h in the morning, calculated from all recorded data during the study period. It became apparent as well that the movement did not take the same time every day. The data on dusk and dawn movement converge towards the equinox on 22nd of September, when the movement is also the fastest. This is explained by the fact that the twilight is also shortest at this time. On the latitude of the Botanical Garden Dresden (51°02′), the difference in nautical twilight between the summer solstice (118 min) and the equinox (71 min) is 47 min (http://cgi.stadtklima-stuttgart.de/mirror/SonneFre.exe). Figure 2 clearly shows that the data around the equinox in September is different from the data around the summer solstice. Thus, not only the duration of the movement is halved, but the standard deviation from 3.07 ± 0.67 h to 1.46 ± 0.35 h as well. Data on the measurements around the winter solstice were neither included in the calculations nor shown in the diagram, as the data must be treated separately. The observed duration of the movement is > 8 h at dusk and ~ 7 h at dawn. Since the length of the day in Dresden is only 8 h at the winter solstice, the rhythm seems disturbed by the strong deviation of the day length from the natural location.

To examine whether particularly short or long durations of movement correlate with the intensity of the light, the latter (Fig. 2) were compared with the data on the average external brightness of the individual days (Fig. 2). For reasons of clarity, the brightness is only shown on days that are also shown in Fig. 2. Particular attention was paid to days with particularly high or low light intensity. For a better impression of the season, the length of the day (time and date AS n.d.) is also shown in Fig. 2.

#### Movement without lamina

Even when the laminae were removed, the plant still performed the nyctinastic movements. The apical ends of the inner petioles peeked out above the water surface. This was especially apparent at night, in the contracted position. The shoot moved slightly downwards in the evening and upwards in the morning, but not as much as with attached laminae.

#### Movement of a single detached leaf

The observation of a detached leaf showed a distinct movement at the base of the petiole (Appendix 2). The upper part of the leaf moved towards the surface of the water. However, after 24 h of separation from the stem, this movement no longer took place.

#### Lighting and darkening experiment

Upon additional illumination with a daylight lamp during the night, after the plant moved into the “sleeping” position as usual in the evening, the rosettes showed no movement, meaning the rosettes did not spread again. When lighting with an assimilation lamp, the plant did not move neither. At dawn, after additional illumination, the rosettes opened as usual.

After covering the aquarium with the box, the plant moved like it would usually do at dusk. After 3 h of darkness the centre of the rosette had gone down. The area of the rosettes decreased by 34% in comparison to the fully spread rosettes before darkening. After 6 h of darkness, it decreased by 39%. After a longer time, the middle of the rosette was still lowered. After 24 h, the rosettes were raised above the water, indicating that the plant’s rhythm was out of sync.

### Morphology and anatomy

*Ludwigia sedoides* is a rooted macrophyte with submersed stems and floating leaves arranged in rosettes that is able to build huge carpets of leaves on the water surface. In the Botanical Garden Dresden, the plant formed rosettes up to a size of 15 cm in diameter. The number of leaves forming the rosette varied. Counting the leaves of 20 different rosettes, resulted in an average number of 72, ranging from 52 to 82. The diameter of the rosettes ranged from 6.5 to 12.3 cm, with an average of 9.27 cm (Appendix 3).

**Fig. 3:**
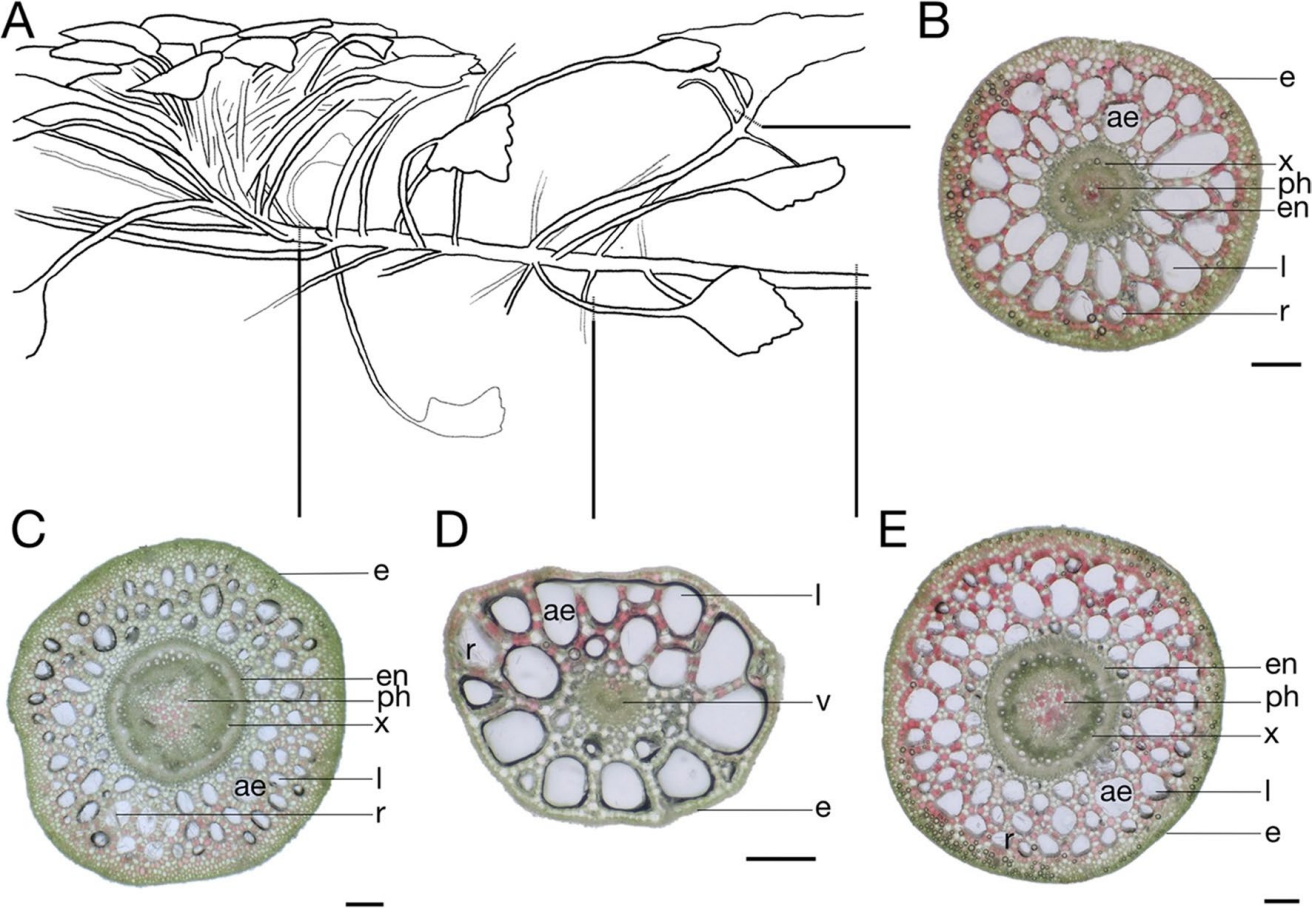
Tissue distribution of *Ludwigia sedoides*. **A** Drawing of *Ludwigia sedoides* sideways with marked positions where the microscopic sections were made. **B** Cross-section side shoot. **C** Cross-section main shoot, 4 cm underneath the rosette. **D** Cross-section petioles. **E** Cross-section main shoot, 16 cm underneath the rosette. Epidermis (e), raphide (r), aerenchyma (ae), lacunae (l), endodermis (en), xylem (x), phloem (ph), vascular bundle (v). Scale bar: 250 µm

In the overview (Fig. 3), a schematic drawing of *L. sedoides* from the side can be seen, as well as the respective cross-sections from the petiole and different parts along the shoot. The shoot axis had a diameter of 3 to 4 mm. The cross-section shows the aerenchyma with huge intercellular spaces. The percentage and size of these spaces increased from the apex to the base of the main shoot. Calcium oxalate raphides were also detected in all four cross-sections. A distinct red coloration of some cells was observed, which formed a kind of checkerboard pattern throughout the cross-section (Appendix 7). The intensity of red coloration was stronger on the upper side of the leaf and at the same time increased towards the base of the shoot. A similar colour structure could be seen in the petiole. It was red coloured on the upper side and mostly green on the lower side. The percentage and size of intercellular spaces of the aerenchyma in the petiole were even larger than in the shoot.

**Fig. 4:**
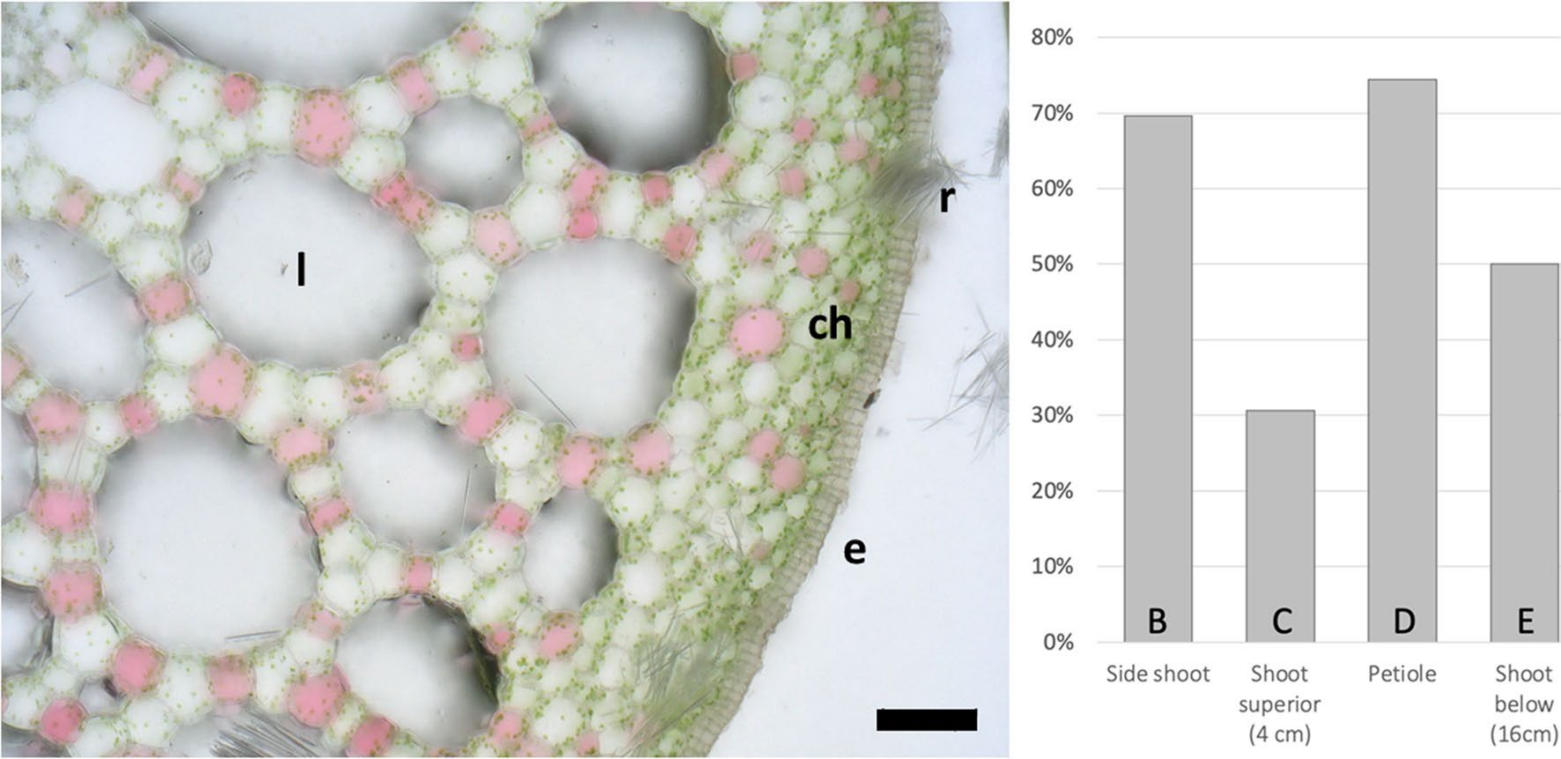
Cross-section of the shoot of *Ludwigia sedoides* (left) and the percentage of the cross-sectional area of intercellular spaces of the aerenchyma (right) in Fig. 3. Chloroplast-rich peripheral tissue (ch), epidermis (e), lacunae (l), raphide (r). Scale bar: 100 µm

The relative amounts of the intercellular spaces of the aerenchyma in different plant parts can be seen in Fig. 4. A proportion of 31% of the cross-sectional area was measured in the upper part of the shoot 4 cm underneath the rosette. Towards basis, 16 cm under the rosette, the proportion comprised 50%. In the aerenchyma of the young side shoot, the area of intercellular spaces sums up to 70% while an even higher percentage of 74% was found in the petiole. Furthermore, numerous chloroplasts could be detected in the shoot (Fig. 4).

**Fig. 5:**
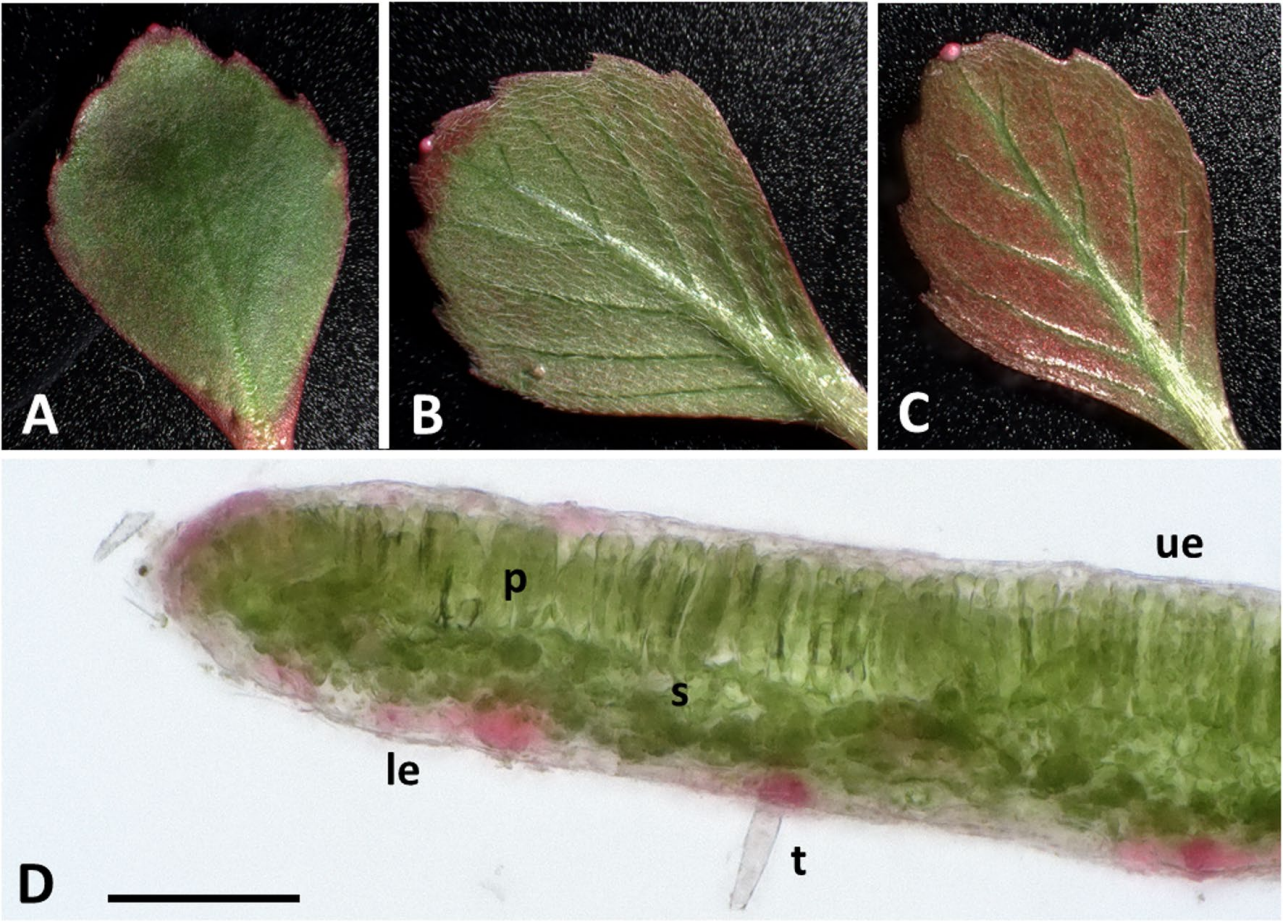
Macroscopic view and cross-section of the lamina of *Ludwigia sedoides*. **A** Upper side of a young leaf lamina. **B** Lower side of a young leaf lamina. **C** Lower side of an old leaf lamina. **D** Cross-section of the lamina. Upper epidermis (ue), lower epidermis (le), palisade parenchyma (p), sponge parenchyma (s), trichome (t). Scale bar: 100 µm

The leaves of *L. sedoides* are usually long-petiolate with a rhombic-ovate, serrate lamina. The laminae of the leaves were mostly green with a red margin. Older leaves became more and more red on the lower side (Fig. 5C). Moreover, short colourless hairs were growing on the lower side of the laminae. The cross-section of the lamina (Fig. 5D) shows the typical structure of a bifacial leaf with upper and lower epidermis, palisade parenchyma and spongy parenchyma. The stomata are positioned on the upper side of the leaf. Furthermore, a red coloration of the cells was visible in the epidermal layers, although this was more pronounced on the abaxial side.

**Fig. 6:**
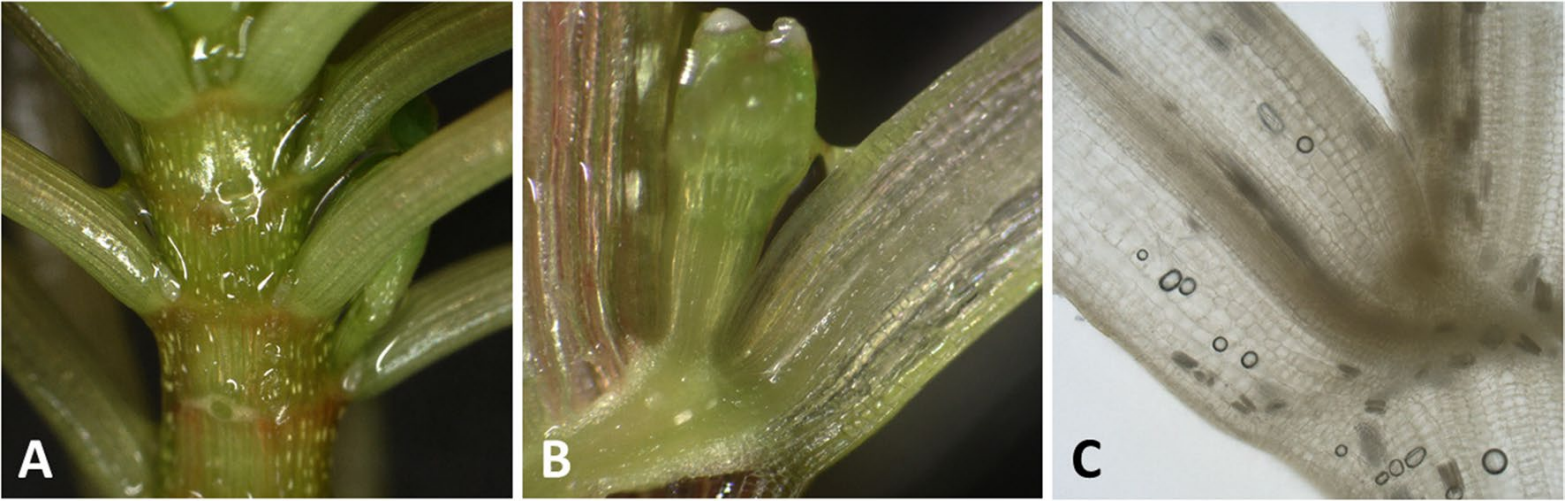
Basal part of the petiole of *Ludwigia sedoides*. **A** Plan view on shoot tip. **B** Longitudinal section, fresh preparation, collected in day position. **C** Longitudinal section, methanol preparation, collected in night position

Both longitudinal and cross-sections were made at the base of the petiole. No joint was recognisable as a separate tissue, forming an obvious pulvinus, neither from the outside nor in a longitudinal section (Fig. 6).

**Fig. 7:**
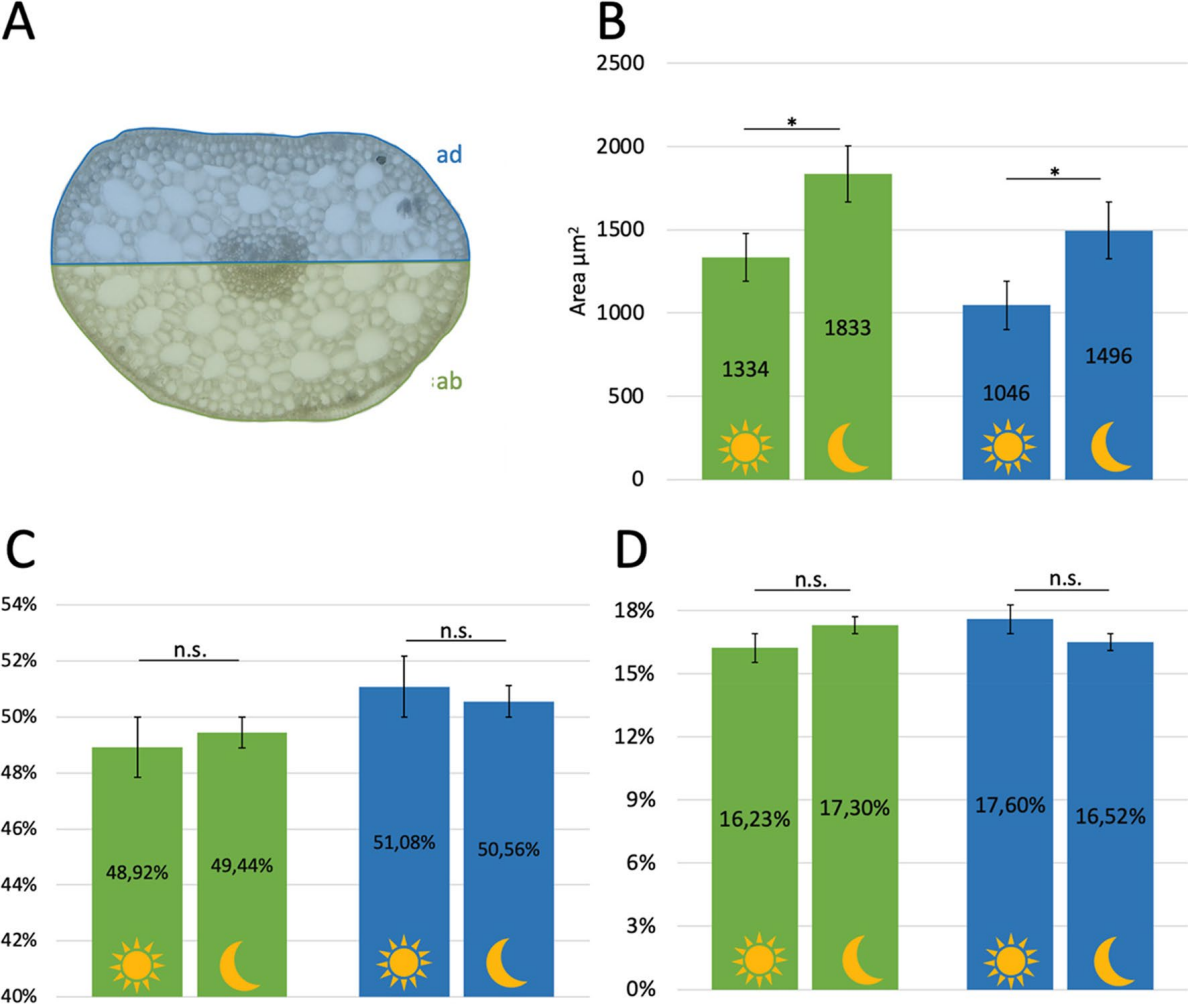
Cross sections measurements of the base of the petiole. **A** Dimensions. **B** Area of individual cells. **C** Area of adaxial (ad) and abaxial side (ab) as shown in **A**. **D** Percentage of intercellular spaces. abaxial (green), adaxial side (blue), daily preparation (sun), nocturnal preparation (moon). not significant (n.s.), significant (*) *P* > 0.05; *P* < 0.05

The cross-sections were made with methanol preparations, from day and night conditions each. An independent two-sample *t*-test was calculated to determine if there are differences in the size of the individual cells on the adaxial and abaxial sides at night and day. The individual cells at the abaxial side were larger both night and day, compared to the adaxial side (Fig. 7B). The difference in the adaxial side was significant (*t* (24) = 6.320, *p* < 0.05) with an average of 1046.40 µm^2^ (standard deviation = 124.09) in day conditions and an average of 1496.32 µm^2^ (s.d. = 224.71) during the night. The difference in the abaxial side was also significant (*t* (24) = 8.242, *p* < 0.05) with an average of 1334.03 µm^2^ (s.d. = 106.62) at daylight and an average of 1833.47 µm^2^ (s.d. = 190.69) at night.

In addition, the area of the abaxial and adaxial sides of the base of the petiole was measured and discussed as proportional numbers in relation to the whole cross section (Fig. 7A). The cross-sectional area on the abaxial side was larger at night with an average of 49.44% (s.d. = 0.012) in comparison to the day with an average of 48.92% (s.d. = 0.015), but there was no statistical significance (Fig. 7C), *U* = 68, *p* < 0.05. In total, the cross-sectional areas of the adaxial side were larger during the day with an average of 51.08% (s.d. = 0.015) in comparison to the night with an average of 50.56% (s.d. = 0.012), but there was no statistically significant difference (Fig. 7C), *U* = 68, *p* < 0.05.

Furthermore, the area of the intercellular spaces was measured and observed as proportion of the adaxial and abaxial cross-sectional areas (Fig. 7D). The difference in the adaxial side was not significant (*t* (24) = 0.674, *p* < 0.05) with an average of 17.35% (s.d. = 4.29%) during the day and an average of 16.11% (s.d. = 5.02%) in the night. The difference in the abaxial side was also not significant (*t* (24) = 0.357, *p* < 0.05) with an average of 16.04% (s.d. = 4.59%) during the day and an average of 16.77% (s.d. = 5.72%) in the night (Appendices 5 and 6).

## Discussion

The results emphasise the complexity of diurnal movements in plants, on the one hand, with regard to the cellular process underlying the movement, and on the other hand, with regard to the procedure of the movement, seen from the outside. This includes the movement’s duration and the dependence on environmental factors. The general observation of the movement by time-lapse videos (Appendix 1) shows differences in the duration of the movement on the measured days. Furthermore, on average, it takes longer in the evening than in the morning on any given day. Studies on circadian oscillators have shown that the influence of light on the pace of the plant clock is incisive and that the quality and intensity of the light affect the plant clock on multiple regulatory levels (Nohales and Kay 2016). On that basis, comparisons of the duration of the movement with weather data from the Botanical Garden were made. The external brightness varies between days due to different weather conditions like variable cloudiness. These fluctuations can also be seen on individual days, so individual peaks can often be observed in the course of the day’s brightness. For this reason, the comparison was based on the average data of the individual days and not on the maximum. But no conspicuous correlations were found.

The movement of *Ludwigia sedoides* depends strongly on the length of the day. In its natural range from Amazonia (0° N) to Mexico (20° N), the day length varies between 11 and 13.5 h. A certain adaptation to this day length is possible for the plant. This is also reflected in the values obtained at the equinox in the study area (51° N). At this time, the day length also varies between 11 and 13.5 h (see Fig. 2). The movement starts even before sunrise, but not before the beginning of civil dawn, which begins when the sun reaches an altitude of − 6° (Seidelmann 2006). This commencement of the movement can therefore be assumed to be the natural one.

With a day length greater than 15 h, the movement starts not before sunrise. With a day length of less than 8 h, the plant seems to be out of rhythm, because the movement starts 6 h before sunrise and lasts 7 h. All these results prove that the plant’s movements are endogenous, although external light factors such as greatly extended or shortened day length or lower lux values due to weather certainly have an influence.

Experiments with permanent darkness and light were conducted to find out whether the movement of *Ludwigia sedoides* could work endogenously. The endogenous circadian clock integrates information of external signals that provide time cues and transfers it to output networks for the regulation of different physiological processes (Greenham and McClung 2015). This allows to synchronise the internal biological rhythm of the plant with the surrounding environment (Inoue et al. 2018). Under nightly illumination, the plant shows no movement of the leaf rosettes. Neither the daylight lamp, nor the assimilation lamp, which illuminated the plant with 25,000 lx, led to a movement out of rhythm at night. Consequently, the movement is not simply triggered by light but rather by an inner clock. In contrast, the plant reacted out of rhythm when it was kept in permanent darkness. It lowered the centre of the rosette, but not as far as during the normal light regime. After a longer period of darkness, it did not lower itself any further. After 1 day of darkness, the rosette rose above the water surface. But this does not appear to be a movement triggered by a circadian oscillator, but rather a reaction to the lack of light, especially considering that the plant needs 11.8 h on average (calculated with the measured data) until it rises again in the morning after starting to move in the evening, whereas in the darkening experiment, the mid of the rosette was still lowered after 18 h. Consequently, the lighting and darkening experiments show contradictory results. With brightness at night, the plant does not react outside its rhythm, which suggests that it is moving under the control of an endogenous oscillator. However, this conclusion does not fit with the darkening experiment, in which the plant lowers itself in the dark but does not reappear. Endogenous characteristics thus seem to play a role as well as environmental factors. This is also supported by the observation around the winter solstice, when the movement already starts 6 h before sunrise.

The microscopic investigations resulted in a better understanding of movement processes. The aerenchyma is an important feature of aquatic plants to survive long-term submergence (Jung et al. 2008). Therefore, it was not surprising to find a well-developed aerenchyma in the shoot and the petioles of *L. sedoides*, classified as a honeycomb aerenchyma (Evans 2004). Raphides could be found mainly in cross-sections of the shoot but also of the petiole. Raphides are needle-shaped calcium oxalate crystals (Raman et al. 2014) that are important for various functions, like the protection from herbivory or intracellular calcium regulation (Nakata 2003). These crystals are present in almost all *Ludwigia* species and are distinctive for Onagraceae in general (Keating 1982).

Since sleep movements can typically be attributed to a pulvinus, a motor cell organ at the base of the petioles (Moran 2007), this part of the plant was particularly interesting for the anatomical investigations. Neither from an external view, nor in longitudinal sections, such a particular tissue at the base of the petiole could be recognised. Nevertheless, the movement originates from this part of the plant, as the video recordings of the individual leaves and of the rosette without lamina show.

Guttenberg (1971) describes the tissue distribution of the pulvini, or so-called variation joints, that allow local leaf movements: The peripherally mounted vascular bundle strands of the leaf petiole unite in the joint to form a central strand, so that bending strength and tensile strength can be ensured. The bending strength, in turn, is usually achieved by increasing the cross-sectional area at the base of the petiole. Since in the aquatic plant *Ludwigia sedoides* the petiole hardly has to bear the weight of the leaf lamina due to buoyancy, less bending strength is required here, so that no typical joint thickening has to be formed at the basal end of the petiole. Furthermore, there are no additional folds on the abaxial side as in other observed species with nyctinastic leaf movements such as *Oxalis rhombifolia* or *Phyllanthus urinaria* (Goebel 1920).

**Fig. 8:**
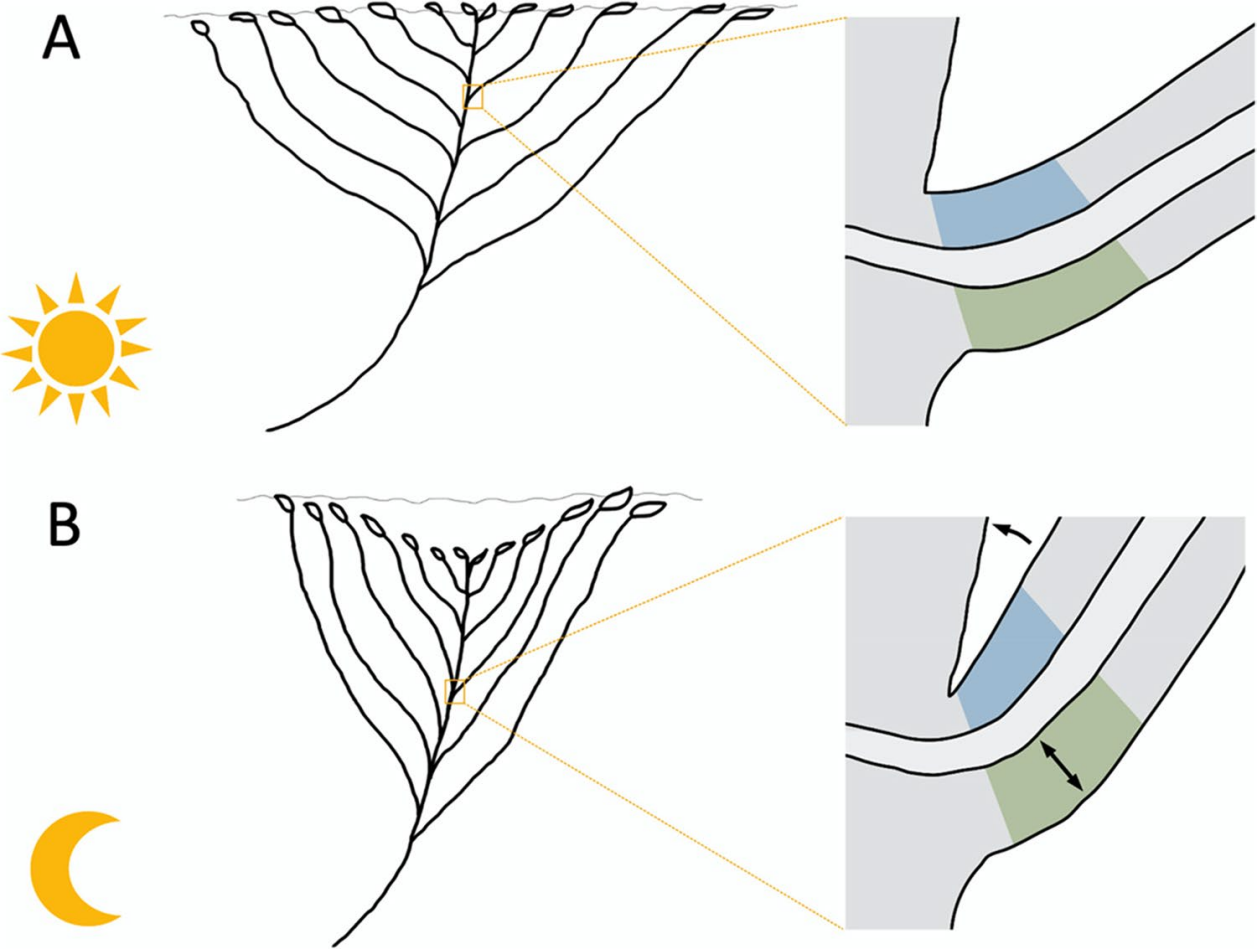
Movement mechanism of the rosette induced by turgor changes in the petiole base. **A** Extended rosette during the day. **B** Submerged position at night. Blue area: adaxial side. Green area: abaxial side

Additionally, the cross-sections of the base of the petiole show significant differences between the measured average size of the individual cells at night and during the day. These could be caused by turgor changes in the respective cells, which allows the movement to be carried out. According to this, the proportions of the abaxial and adaxial sides of the cross-sections of the bottom of the petiole were measured. The average of the adaxial sides in the sections by day (51.08%) was bigger than the adaxial sides in the nocturnal Sects. (50.56%), whereas the abaxial side is bigger in the nocturnal Sects. (49.44%) than in the sections by day (48.92%). A conception of how this enlargement could cause the movement is sketched in Fig. 8. Through the swelling of the abaxial side of the base of the petiole through an increase of turgor pressure, the leaf moves closer to the shoot and the rosette closes (Fig. 8B). This causes the rosette to contract and the diameter to decrease. Because the laminae of the outer leaves remain on the water surface, the centre of the rosette may sink, as shown by the experiment with removed laminae. The submergence of the central leaves of the rosette, instead of lifting the outer leaves above the water level, can be explained by the interfacial tensions acting between leaves, water and surrounding air, respectively. The laminae lie on the water surface and therefore decrease the water–air interface, which is energetically favourable. Raising the leaves above the water level would require energy to create an additional water–air interface, which corresponds to a force needed to pull the leaves above the water. This force obviously is larger than the buoyancy caused by the aerenchyma due to the large surface area of the leaf laminae and consequently results in a downward movement of the central part of the rosette. This explanation is supported by the observation that the petioles peek out above the water surface, when the laminae are removed. The rosette spreads again when the abaxial side is shrinking through a loss of turgor pressure and the adaxial side is swelling (Fig. 8A). The measured differences on both sides are not statistically significant but could be large enough to cause the plant to move. The principle of the pulvinus is turgor changes of the flexor motor cells and the extensor motor cells (Ueda et al. 2019). The extensor cells gain turgor and increase in size and the flexor cells lose turgor and shrink during the opening of the leaf (Gorton and Satter 1983). Even if there is no swollen base as typical for a pulvinus, *L. sedoides* seems to manage the movement with a similar mechanism: changes in the size of opposing tissues through turgor changes.

## Conclusion

The studies on *Ludwigia sedoides* successfully documented their sleep movements for the first time. Endogenous factors play a decisive role, with environmental factors such as day length or cloud cover exerting a further influence. Withal, the base of the petiole represents the decisive organ, even if it is not thickened, as in typical pulvini.

It would also be interesting to investigate the benefit of the foliar nyctinasty for this aquatic plant species. Furthermore, examinations of cellular processes, like measurements of the turgor pressure in the petiole, would help to further understand the mechanism. Molecular and chemical investigations could possibly also explain why the movement is usually faster in the morning than in the evening.

## Supplementary Information

Below is the link to the electronic supplementary material.

Appendix 1: Time Lapse Videos. (MP4 61.1 MB).

Time Lapse Videos. (MP4 41.3 MB).

Time Lapse Videos. (MP4 24 MB).

Appendix 2: Video Movement of single leaf (MP4 16.8 MB)

Appendix 3: Diameter of rosettes and number of leaves. (DOCX 13.9 KB)

Appendix 4: Data of weather station. (XLSX 386 KB)

Appendix 5: Cross section measurements of the base of the petiole. Area of individual cells as mean value for each cross section. (XLSX 10.5 KB)

Appendix 6: Cross section measurements of the base of the petiole. Area of adaxial and abaxial side and of intercellular spaces. (XLSX 11 KB)

Appendix 7: Thin Layer Chromatography and Spectrophotometric Detection. (DOCX 389 KB)
