# Supplementary material for: Morphology, anatomy and sleep movements of Ludwigia sedoides
Source: Naturwissenschaften. 2023 May 15;110(3):18. doi: 10.1007/s00114-023-01848-7 (PMC10185591; doi:10.1007/s00114-023-01848-7)
Supplement: Supplementary file 5 — Appendix 3: Diameter of rosettes and number of leaves. (DOCX 13.9 KB) [file 114_2023_1848_MOESM5_ESM.docx]

| **Appendix 3:** Diameter of rosettes and number of leaves. | |
| --- | --- |
|  |  |
| **Diameter of the rosette [cm]** | **Number of leaves** |
| 9.7 | 78 |
| 6.7 | 53 |
| 9.1 | 65 |
| 9.9 | 79 |
| 6.5 | 69 |
| 9.0 | 81 |
| 9.2 | 68 |
| 8.4 | 66 |
| 9.0 | 79 |
| 9.7 | 76 |
| 10.4 | 82 |
| 9.2 | 67 |
| 6.8 | 52 |
| 9.3 | 63 |
| 12.3 | 82 |
| 8.1 | 76 |
| 6.5 | 63 |
| 11.8 | 76 |
| 11.6 | 79 |
| 9.8 | 69 |
| **9.2** | **71.2** |
