# Supplementary material for: Morphology, anatomy and sleep movements of Ludwigia sedoides
Source: Naturwissenschaften. 2023 May 15;110(3):18. doi: 10.1007/s00114-023-01848-7 (PMC10185591; doi:10.1007/s00114-023-01848-7)
Supplement: Supplementary file 9 — Appendix 7: Thin Layer Chromatography and Spectrophotometric Detection. (DOCX 389 KB) [file 114_2023_1848_MOESM9_ESM.docx]

## Appendix 7: Thin Layer Chromatography and Spectrophotometric Detection

A red colour is found in the epidermal layers of the lamina, mainly in the lower epidermis and also in the shoot and the petiole. In a cross-section of the shoot, the red coloured cells are evenly distributed. The spectrophotometric detection (**Fig. 10**) was done with the four upper bands (**Fig. 9**).


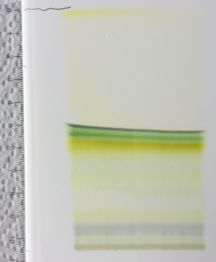


Figure 9: Thin layer chromatography plate. Arrows point to bands, used for the spectrophotometric detection.


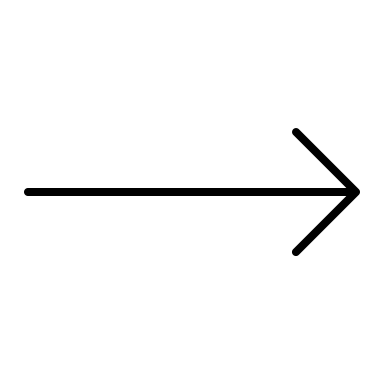

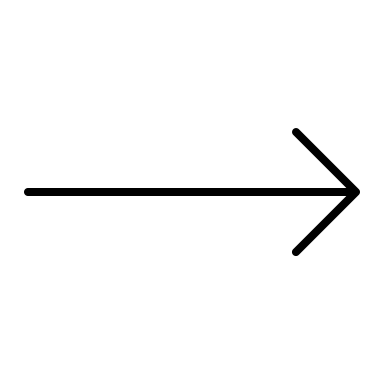

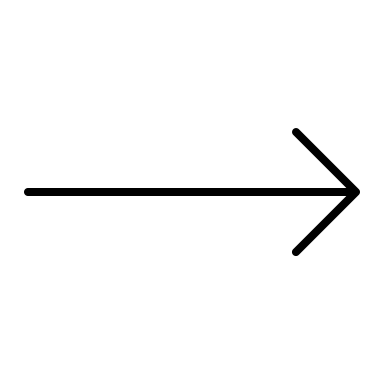

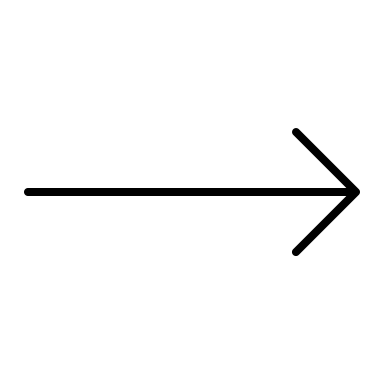


1

3

2

4

The yellow first top band did not show a clear and evaluable spectrum. The second and dark green band show maxima at 410 nm and 667 nm, the third and brighter green band show maxima at 455 nm and 645 nm and the fourth and more intense yellow band show three maxima at 427 nm, 446 nm and 471 nm. Chlorophyll absorbs light in the range from 400 to 800 nm and 550 to 700 nm (Kadereit et al., 2014). With the mentioned measurements and the consideration of the position of the bands on the plate, the dark green band can be identified as chlorophyll a, and the brighter green band as chlorophyll b. Carotenoids absorb light from about 460 to 500 nm (Kadereit et al., 2014). Considering the position on the plate of the thin layer chromatography, the third and intense yellow coloured band can be identified as Lutein (Wagner, 2009).


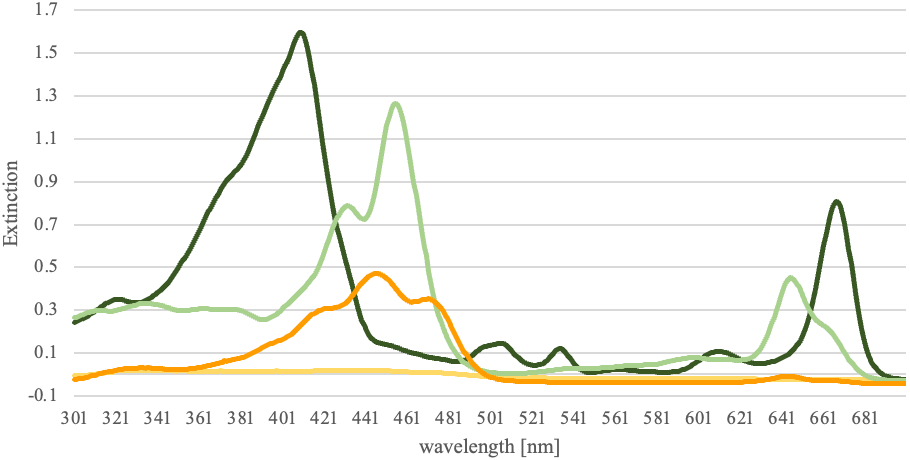


Figure 10: Spectra of the spectrophotometric detection based on the thin layer chromatography. **Yellow** first top band (Fig. 9.1), not evaluable. **Dark green** second band (Fig 9.2), identified as chlorophyll a. **Light green** third band (Fig. 9.3), identified as chlorophyll b. **Orange** fourth band (Fig. 4.4)**,** identified as lutein.

References:

Kadereit JW, Körner C, Kost B, Sonnewald U (2014) Lehrbuch der Pflanzenwissenschaften (Strasburger E, Noll EF, Schenck H, Schimper AFW, Eds.; 37. Auflage). Springer Spektrum.

Wagner, W. (2009). Chromatographie und Aufnahme von Absorptionsspektren der Blattfarbstoffe grüner Blätter. <http://daten.didaktikchemie.uni-> [bayreuth.de/experimente/](http://bayreuth.de/experimente/) chembox/u_blattfarbstoffe/blatt_extinktion.htm
